# Exploring the immunomodulatory role of depot medroxyprogesterones acetate and endogenous progesterone levels in HIV infected and uninfected women

**DOI:** 10.1186/s13104-019-4785-z

**Published:** 2019-11-15

**Authors:** Nonzwakazi Mnqonywa, Nathlee Abbai, Viswanath Ragupathy, Gita Ramjee, Indira Hewlett, Dhayendre Moodley

**Affiliations:** 1 HIV Prevention Research Unit, Medical Research Council, 123 Jan Hofmeyr Road, Westville, Durban, 3630 South Africa; 2 School of Clinical Medicine Research Laboratory, University of KwaZulu-Natal, 719 Umbilo Road, Congella, 4013 South Africa; 3 Center for Biologics Evaluation and Research, Food and Drug Administration, Building 72, 10933 New Hampshire Ave, Silver Spring, MD 20892 USA; 4 Department of Epidemiology and Population Health, London School of Hygiene and Tropical Medicine, London, UK; 5 Women’s Health and HIV Research Unit, Department of Obstetrics and Gynaecology, School of Clinical Medicine, University of KwaZulu-Natal, 719 Umbilo Road, Congella, 4013 South Africa

**Keywords:** Depot medroxyprogesterone acetate (DMPA), Human immunodeficiency virus (HIV), Gene expression, Chemokine receptor type 5 (CCR5), Immunomodulatory, Endogenous progesterone

## Abstract

**Objective:**

The aim of this proof of concept study was to determine the effect of depot medroxyprogesterone acetate on host and viral factors in HIV infected and uninfected women.

**Results:**

In this study, the gene expression levels for CCL5, CCR5 and CXCR4 was significantly higher in HIV positive women when compared to HIV negative women (p < 0.05). An upregulation of CCR5 and CXCR4 was evident in less than 20% of the HIV infected women and none of the HIV uninfected women. The mean fold change for CCL3 was much higher in HIV uninfected when compared to infected women with a borderline significance (p = 0.062). In HIV uninfected women, the mean fold change in CCL3, CCL4, and CCL5 gene expression was not statistically different between women on DMPA versus women not on hormonal contraception. The proportion of women with an upregulation of CCL4 and CCR5 was higher in HIV infected women on DMPA. There was no association between endogenous progesterone level and chemokines and the HIV-1 receptors. The gene expression levels in the chemokine receptors CCR5 and CXCR4 were significantly higher in the HIV infected women when compared to the women who remained HIV uninfected.

## Introduction

Since its introduction as a safe long acting contraceptive method in 1949, an estimated 50 million women worldwide use the progestin-only depot-medroxyprogesterone acetate (DMPA) injectable [1]. In sub-Saharan Africa alone, an estimated 60% of the 14 million women who use hormonal contraception choose to use the 3-monthly injectable, DMPA [2]. In high Human Immunodeficiency virus (HIV) burden countries, increased contraceptive use among HIV infected women has indirectly reduced the number of HIV exposed and infected children [3].

In a meta-analysis of several epidemiological studies, the adjusted hazards ratio for HIV risk among DMPA users varied between 0.46 and 2.84 [4]. According to Smith-McCune et al. [5] and Ghanem et al. [6] hormonal contraception may increase susceptibility to HIV-1 infection via the following mechanisms; increasing inflammatory responses which result in the activation and recruitment of target cells for HIV-1 thereby increasing viral replication; negating host defensives by decreasing the secretion of antimicrobial peptides that contribute to host defensives; increasing the expression of HIV-1 co-receptors; thinning of the epithelial barrier resulting in changes in the vaginal microbiome; increasing the genital shedding of and risk for herpes; and increasing the frequency of cervical HIV target cells (CCR5 + and CD4 + T cells).

In a young non-pregnant South African cohort, both DMPA and a high level of endogenous progesterone during the luteal phase of the menstrual cycle were strongly associated with a localization of HIV target cells in the cervix [7].

In a small group of HIV infected and uninfected women we sought to explore the effect of DMPA and high endogenous progesterone levels on chemokine and chemokine receptor gene expression that are thought to increase susceptibility to HIV acquisition and disease progression.

## Main text

### Study setting and population

This was a cross-sectional proof of concept study conducted in collaboration with the Center for Biologics Evaluation and Research, Food and Drug Administration, Maryland, USA. Twenty HIV infected and 20 HIV uninfected non-pregnant women were enrolled at the Tongaat research site of the South African Medical Research Council, HIV Prevention Research Unit (SAMRC-HPRU) in Durban between November and December 2015. The HIV infected women were antiretroviral treatment naïve. It was only in September 2016, that South Africa launched the “Test and Treat” program and all HIV infected persons could access antiretroviral treatment irrespective of their CD4 count.

For each of the HIV infected and uninfected groups, we enrolled 10 women who reported DMPA use and 10 women who were not taking any hormonal contraception per group. Contraception use was self-reported and subsequently verified by examining their family planning card.

### Sample collection

Women were eligible for study participation if they were between the ages of 18–45 years old, willing to provide written informed consent, willing to provide a blood sample and able to provide reliable records pertaining to family planning. Pregnant women and women on active antiretroviral therapy for HIV, were excluded from the study.

The enrolled women provided 30 ml (3 × 10 ml) Ethylenediaminetetraacetic acid (EDTA) venous whole blood samples, collected by a trained phlebotomist. The blood was separated into plasma and peripheral blood mononuclear cell (PBMCs) fractions. Plasma was used for determining the level of progesterone and PBMCs were used for the gene expression assays.

### Determination of progesterone levels

Progesterone levels were determined using the VIDAS® Progesterone (PRG) assay. A total of 200 µl of plasma was used for testing. The VIDAS® Progesterone (PRG) assay is an enzyme-linked fluorescent immunoassay (ELIFA). The measurement range of the VIDAS Progesterone reagent was 0.25–80 ng/ml. Progesterone values below the limit of detection (<0.25 ng/ml) were read as half the limit of detection (i.e. 0.125 ng/ml. A standard and control were run once a day (Package Insert, VIDAS® Progesterone (PRG).

### Isolation of peripheral blood mononuclear cells (PBMCs)

The Ficoll density gradient separation method of whole blood was used for separation of mononuclear cells. Purified PBMCs (± 5 × 10^6^) were frozen in liquid nitrogen and stored immediately at − 150 °C. The PBMC samples were shipped under appropriate conditions to the Center for Biologics Evaluation and Research, Food and Drug Administration, United States. Viable PBMCs were enumerated using the Invitrogen Countess Automated Cell Counter (Cat No: C10227), cells stained with trypan blue were considered as dead cells. The study enrolled 40 women. Although PBMCs were isolated soon after blood collection and frozen in liquid nitrogen, an adequate yield of viable PBMCs that provided RNA of high integrity was only available for 28 of the 40 samples (15/20 HIV infected and 13/20 HIV uninfected women).

### RNA purification and cDNA synthesis

The RNeasy® Plus Mini kits for RNA purification from PBMCs were used as per the kit insert. The RNA was quantified using the NanoDrop One instrument (Thermo Scientific). The RT2 Easy First Strand Kit was used to provide a convenient and rapid method for efficient first strand cDNA synthesis. The cDNA mix was stored at − 20 °C until further use.

### Gene expression analysis

The gene expression assays were conducted in 96-well plates containing primer assays for 84 pathways or disease focused genes and 5 housekeeping genes. The 84 genes were not specifically selected for this study but were part of a predesigned commercially available kit (RT2 Profiler PCR Array, QIAGEN). Cycling conditions used were as followed: 1 cycle at 95 °C for 10 min followed by 40 cycles at 95 °C for 15 sec and 60 °C for 1 min. The final relative expression (data) was then interpreted using the Cycling threshold (CT) values obtained.

### Data management and statistical analysis

The laboratory data for this study was analysed using an easy-to-use Excel-based data analysis template or Web-based software. Data analysis was based on the CT method with standardization of the raw data to the housekeeping genes. All the CT values reported as greater than 35 or as not detected/NA were changed to 35 and the values equal to 35 were considered a negative call.

Fold change results were interpreted in a biological meaningful way and fold change values greater than two (> 2) indicated a positive or an up regulation. Fold change values less than two (< 2) indicated a negative or down regulation. The fold regulation cut-off was equal to two. P values were calculated based on a student t-test of the replicate of Delta CT values for each gene in the control group as well as the treatment groups. The p values cut-off was 0.05. The genomic DNA contamination was regarded as passed when the CT was greater or equal to 35 (CT ≥ 35). Epi Info statistical software was used to calculate significance.

## Results

### Regulation of inflammatory markers and HIV receptors in association with HIV status

Although 84 genes were analysed, we focused on select inflammatory markers previously reported to be associated with HIV infection i.e. CCL3, CCL4 and CCL5, CCR5 and CXCR4. The mean fold change in CCL5, CCR5 and CXCR4 was significantly higher in HIV infected women when compared to HIV uninfected women (Table 1). Mean fold change in CCL3 on the other hand was much higher in HIV uninfected women but of borderline significance (p = 0.062). The majority (85%) of HIV uninfected women demonstrated an upregulation of CCL3 (>twofold change) in comparison to 50% of HIV infected women (p = 0.086). An upregulation of CCR5 and CXCR4 was evident in less than 20% of the HIV infected women and none of the HIV uninfected women.

**Table 1:** Mean (SD) fold change in chemokines and chemokine receptors in HIV infected and uninfected women

	HIV infected women (n = 15)	HIV uninfected women (n = 13)	p value
CCL3	2.68 (2.10)	3.92 (2.09)	0.062
CCL4	2.85 (1.95)	3.06 (5.01)	0.160
CCL5	2.77 (1.85)	1.15 (1.59)	0.005
CCR5	1.41 (0.59)	0.67 (0.34)	0.0003
CXCR4	1.26 (0.54)	0.46 (0.52)	0.001

### Effect of DMPA on regulation of inflammatory markers and HIV receptors

In the HIV infected women there was no significant difference in level of expression for CCL3, CCL4, CCL5, CCR5 and CXCR4 in DMPA users versus non-hormonal contraception (NHC) users (p > 0.05) (Table 2). Similarly, there was no significant difference in the expression levels of CCR5 and CXCR4 across DMPA and NHC users (p > 0.05). In the HIV uninfected group, there was also no significant difference in level of expression for CCL3, CCL4 and CCL5 in DMPA users versus NHC users (p > 0.05). CCR5 and CXCR4 were downregulated across both groups, with a borderline significance (p = 0.057) for CXCR4.

**Table 2:** Mean (SD) fold change in chemokines and chemokine receptors in HIV infected and uninfected women stratified by DMPA exposure

	HIV infected women (n = 15)			HIV uninfected women (n = 13)		
	DMPA, (N = 7)	Not on hormonal contraception, (N = 8)	p value	DMPA, (N = 8)	Not on hormonal contraception, (N = 5)	p value
CCL3	3.36 (2.85)	2.08 (1.02)	0.418	4.63 (2.28)	2.78 (1.14)	0.107
CCL4	3.81 (2.27)	2.00 (1.21)	0.083	3.49 (6.26)	2.37 (2.39)	0.661
CCL5	2.96 (2.03)	2.60 (1.81)	0.908	1.26 (2.00)	0.98 (0.76)	0.306
CCR5	1.69 (0.74)	1.17 (0.29)	0.133	0.58 (0.36)	0.80 (0.27)	0.188
CXCR4	1.25 (0.59)	1.26 (0.53)	0.817	0.22 (0.23)	0.83 (0.65)	0.057

### Effect of endogenous progesterone on regulation of inflammatory markers and HIV receptors

For CCL3, a higher endogenous progesterone level (EPL) was measured in women who had a ≥ twofold upregulation when compared to women who had a < twofold regulation (p = 0.884) (Table 3). For CCL4, a higher EPL was measured in women who had a two-fold upregulation (p = 0.714). For CCL5, a higher EPL was observed for women who had < twofold change for this chemokine. Similar to CCL3 and CCL4, there was no significant association between the level of expression and EPL for CCL5 (p = 0.519). Since there were no women with an upregulation of CCR5 and only one for CXCR4, a similar comparison in progesterone levels could not be done however the mean and SD of progesterone levels are included in the table.

**Table 3:** Mean (SD) plasma endogenous progesterone level (ng/µl) in Association with ≥ 2 fold change in chemokine and chemokine receptors in women not on hormonal contraception

	≥ 2 fold change (n = 8)	< 2 fold change (n = 5)	p value
CCL3	2.41 (2.92)	0.68 (2.09)	0.884
CCL4	2.34 (2.64)	1.37 (5.01)	0.714
CCL5	1.19 (1.78)	2.23 (2.88)	0.519
CCR5	–	1.75 (2.40)	–
CXCR4	4.8 (n = 1)	1.49 (2.32)	0.284

## Discussion

Chemokine receptors CCR5 and CXCR4 and their natural ligands CCL3, CCL4 and CCL5 have been identified as prominent markers of HIV acquisition and viral replication. Defect in CCR5 gene expression in particular has been postulated as a significant determinant of individuals remaining uninfected despite repeat exposure to HIV-1 [8]. The role of progestin containing contraception particularly DMPA in HIV acquisition and accelerated disease progression has been much debated in recent years. Endogenous progesterone itself is thought to increase susceptibility to HIV acquisition and increase viral replication during heightened levels in the menstrual cycle [7]. In this proof of concept study we report levels of mRNA gene expression of CCL3, CCL4, CCL5 and CCR5 and CXCR4 in association with HIV-1 infection, DMPA exposure and endogenous progesterone level in a small group of women who were infected during a HIV prevention trial and a group of sexually active women who remained HIV uninfected well after the HIV prevention study ended.

In vitro studies have shown that HIV receptors such as CD4 + , CCR5 and CXCR4 are regulated by sex hormones [9, 10]. These studies performed with activated PBMC cultures showed that incubation with medroxyprogesterone acetate (MPA) increases HIV-1 replication and prevents downregulation of HIV co-receptors on CD4 + T cells. In addition, immune cells from vaginal biopsies of women using DMPA have also been shown to express high levels of CCR5 [5]. In the current study there is evidence of the higher fold upregulation of CCR5 in HIV infected women taking DMPA when compared to women who were not on hormonal contraception, however not statistically significant. Similarly, a higher gene expression level in CCL3, CCL4 and CCL5 was observed for the HIV infected women on DMPA when compared to women not on hormonal contraception.

Other pro-inflammatory chemokines CCL3, CCL4 and CCL5 have been shown to suppress HIV-1 replication by inhibiting viral replication and binding CCR5, one of the primary co-receptors that HIV-1 utilizes for entry into CD4 T cells [11]. In the current study, we observed a higher level of expression of CCL3 and CCL4 in the HIV uninfected women when compared to the infected women. According to Bugeja et al. [12], patients repeatedly exposed to, but uninfected by HIV-1 secrete higher levels of the CCR5, CCL3, CCL4 and CCL5 suggesting that these chemokines may also confer natural resistance to infection. In the current study, within the HIV uninfected women; CCL3, CCL5 and CCL4 were differentially upregulated in the presence of DMPA. However, CCR5 and CXCR4 were not upregulated. A previous study had reported that peripheral CD4 + CCR5 expression was absent in HIV uninfected women on DMPA [13].

In this study, the gene expression levels of CCR5 and CXCR4 were significantly higher in the HIV infected women when compared to the women who remained HIV uninfected. Another important finding, was the significantly higher upregulation of CCL3 in HIV uninfected women. When stratified by DMPA use, the level of gene expression for CCR5, CXCR4, CCL3, CCL4 and CCL5 was not associated with the presence of DMPA. Neither was there an association between endogenous progesterone level and chemokines and the HIV-1 receptors.

## Limitations

This study did not explore an association between chemokine expression and HIV-1 viral load or CD4 count due to the limited size of the study population. Despite limitation, some of the findings presented in this study are supported by recently published studies.

## Data Availability

The data generated in this study is available from the corresponding author.

## Abbreviations

CT: cycling threshold
DMPA: depot-medroxyprogesterone acetate
EDTA: ethylenediaminetetraacetic acid
EPL: endogenous progesterone level
HIV: human Immunodeficiency virus
NHC: non-hormonal contraception
MPA: medroxyprogesterone acetate
PBMCs: peripheral blood mononuclear cells

## References

[1] Morrison CS, Skoler-Karpoff S, Kwok C, Chen P-L, van de Wijgert J, Gehret-Plagianos M, Patel S, Ahmed K, Ramjee G, Friedland B (2012). Hormonal contraception and the risk of HIV acquisition among women in South Africa. AIDS..

[2] McCoy SI, Zheng W, Montgomery ET, Blanchard K, van Der Straten A, de Bruyn G, Padian NS (2013). Oral and injectable contraception use and risk of HIV acquisition among women in sub-Saharan Africa. AIDS..

[3] Petruney T, Robinson E, Reynolds H, Wilcher R, Cates W (2008). Contraception is the best kept secret for prevention of mother-to-child HIV transmission. Bull World Health Organ.

[4] Polis CB, Curtis KM (2013). Use of hormonal contraceptives and HIV acquisition in women: a systematic review of the epidemiological evidence. Lancet Infect Dis.

[5] Smith-McCune K, Hilton J, Shanmugasundaram U, Critchfield J, Greenblatt R, Seidman D, Averbach S, Giudice L, Shacklett B (2017). Effects of depot-medroxyprogesterone acetate on the immune microenvironment of the human cervix and endometrium: implications for HIV susceptibility. Mucosal Immunol..

[6] Ghanem KG, Shah N, Klein RS, Mayer KH, Sobel JD, Warren D, Jamieson DJ, Duerr AC, Rompalo AM, Group HERS (2005). Influence of sex hormones, HIV status, and concomitant sexually transmitted infection on cervicovaginal inflammation. J Infect Dis.

[7] Byrne EH, Anahtar MN, Cohen KE, Moodley A, Padavattan N, Ismail N, Bowman BA, Olson GS, Mabhula A, Leslie A (2016). Association between injectable progestin-only contraceptives and HIV acquisition and HIV target cell frequency in the female genital tract in South African women: a prospective cohort study. Lancet Infect Dis.

[8] Trecarichi EM, Tumbarello M, de Gaetano DK, Tamburrini E, Cauda R, Brahe C, Tiziano FD (2006). Partial protective effect of CCR5-Delta 32 heterozygosity in a cohort of heterosexual Italian HIV-1 exposed uninfected individuals. AIDS Res Ther..

[9] Ragupathy V, Devadas K, Tang S, Wood O, Lee S, Dastyer A, Wang X, Dayton A, Hewlett I (2013). Effect of sex steroid hormones on replication and transmission of major HIV subtypes. J Steroid Biochem Mol Biol..

[10] Ragupathy V, Xue W, Tan J, Devadas K, Gao Y, Hewlett I (2016). Progesterone augments cell susceptibility to HIV-1 and HIV-1/HSV-2 co-infections. J Mol Endocrinol.

[11] Hudspeth K, Fogli M, Correia DV, Mikulak J, Roberto A, Della Bella S, Silva-Santos B, Mavilio D (2012). Engagement of NKp30 on Vδ1 T cells induces the production of CCL3, CCL4, and CCL5 and suppresses HIV-1 replication. Blood.

[12] Bugeja MJ, Booth DR, Bennetts BH, Guerin J, Kaldor JM, Stewart GJ (2004). Analysis of the CCL3-L1 gene for association with HIV-1 susceptibility and disease progression. AIDS..

[13] Sciaranghella G, Wang C, Hu H, Anastos K, Merhi Z, Nowicki M, Stanczyk FZ, Greenblatt RM, Cohen M, Golub ET (2015). CCR5 expression levels in HIV-uninfected women receiving hormonal contraception. J Infect Dis.

